# Microfluidic Technology for Clinical Applications of Exosomes

**DOI:** 10.3390/mi10060392

**Published:** 2019-06-12

**Authors:** Florina S. Iliescu, Danilo Vrtačnik, Pavel Neuzil, Ciprian Iliescu

**Affiliations:** 1 School of Applied Science, Republic Polytechnic Singapore, Singapore 738964, Singapore; florina_iliescu@rp.edu.sg; 2 Laboratory of Microsensor Structures and Electronics, Faculty of Electrical Engineering, University of Ljubljana, SI-1000 Ljubljana, Slovenia; danilo.vrtacnik@fe.uni-lj.si; 3 Ministry of Education Key Laboratory of Micro/Nano Systems for Aerospace, School of Mechanical Engineering, Northwestern Polytechnical University, Xi’an 710072, China; 4 Central European Institute of Technology, Brno University of Technology, Brno 613 00, Czech Republic; 5 Department of Microelectronics, Faculty of Electrical Engineering, Brno University of Technology, Technická 3058/10, 61600 Brno, Czech Republic; 6 Biomedical Institute for Global Health Research and Technology (BIGHEART), National University of Singapore, Singapore 117599, Singapore; 7 Academy of Romanian Scientists, Bucharest 050094, Romania

**Keywords:** microfluidics, exosomes, clinical applications, extracellular vesicles, cancer, inflammation

## Abstract

Exosomes, a type of nanovesicle, are distinct cellular entities specifically capable of carrying various cargos between cells. It has been hypothesized that exosomes, as an enriched source of biomolecules, may serve as biomarkers for various diseases. This review introduces general aspects of exosomes, presents the challenges in exosome research, discusses the potential of exosomes as biomarkers, and describes the contribution of microfluidic technology to enable their isolation and analysis for diagnostic and disease monitoring. Additionally, clinical applications of exosomes for diagnostic purposes are also summarized.

## 1. Introduction

The transport of biological material across membranes in the form of microparticles and well-compartmented nanovesicles is an essential mechanism, conserved throughout the evolution pathways of cellular homeostasis, physiologically allowing cells to actively or passively export micro- and macromolecules [1]. However, when homeostasis is disrupted, causing pathological changes, an aberrant export of either key proteins or nucleic acids occurs, leading to their aberrant expression. The increased release of nanovesicles and their accumulation appear to be important in pathological transformation processes such as cancer and inflammatory-based diseases. Exosomes belong to the category of compartmented extracellular vesicles, and are the smallest of them all, with unique biogenesis. The existence of extracellular vesicles was suggested in 1946 [2], and their isolation and specific nomenclature subsequently followed [3]. Dedicated studies have described in increasing detail these ubiquitous features of viable cells, often named membrane fragments, platelet dust, or cellular debris [4]. Currently, it is generally accepted that exosomes are small in range (from tens to hundreds of nm), and are lipid bilayer membrane nanovesicles of endocytic origin with a highly selective and variable content, secreted by nearly all cells both in vitro and in vivo and involved in intercellular communication [3,5]. Exosomes have a few morphological characteristics in common: size, density, shape, general lipid or protein composition, and subcellular origin [3,6,7,8,9]. However, the observation that the exosomes’ cargo changes depending on the cell of origin and the physiological and pathological conditions [10,11,12,13,14], and that they are easily accessible in practically all human biofluids, has triggered intense research into their possible use as non-invasive biomarkers. Furthermore, the prognosis of diseases depends on an examination of the affected tissues, via a tissue or liquid biopsy, that provides complementary information to enable an accurate diagnosis and disease monitoring. Compared with a tissue biopsy, considered the gold standard in histopathology, a liquid biopsy is a non-invasive procedure that samples biofluids, provides information on various pathologies, and has the potential to provide real-time feedback on a patient’s condition from diagnosis to therapy monitoring.

However, providing critical information regarding the biomarkers requires diagnostic tools with a high sensitivity and specificity. Current standard technology for isolating exosomes is differential centrifugation. Despite the high purity of the product, it is time-consuming, expensive, and inefficient [15]. Another method for the analysis of microvesicles is flow cytometry. It offers a high throughput and molecular detection ability, but its performance is limited by weak light scattering at the vesicles as their size is significantly smaller than the wavelength of utilized light [16]. Consequently, combinations of methods have been proposed to increase the accuracy of detection, isolation for further analysis, and characterization. Recently, microfluidic methods were developed in order to isolate exosomes from whole blood in a label-free and contact-free manner [17]. 

Here, we present recent advances of the microfluidic platforms for the detection, isolation, and characterization of exosomes. The proposed platforms offer several advantages in terms of simplicity of the protocols, smaller sample volumes, a lower concentration of exosomes for analysis, a higher throughput, and on-chip combined assays. The work also reviews the present clinical status of exosomes related to microfluidic technology It is accepted that exosomes offer significant advantages for cancer diagnostics and monitoring, in terms of their abundance, stability, and diversity, as well as their identification in large quantities in a variety of bodily fluids, including blood, ascites, and cerebrospinal fluid. However, it has been acknowledged that the translation of exosomes towards clinical application and Point-of-Care testing (POCT) poses unique sets of challenges, primarily because of the small dimension of exosomes and the incomplete understanding of their biology and subsequently because of the difficulty in their isolation and detection. Therefore, perfecting the on-chip isolation and analysis methods for integrated microfluidic platforms is crucial. 

## 2. General Aspects of Exosomes

### 2.1. Genesis 

After Johnstone isolated and described exosomes as endosome-derived vesicles [3], other mechanisms started to be explored to further depict the processes involved in the selective degradation or diminishing of the cells’ protein “menu” of the plasma membrane [18,19,20,21]. In eukaryotic cells, they form intracellularly, as intraluminal vesicles of the multivesicular body (MVB) and early endosomes. The buddying consists of invagination of the limiting membrane of early endosomes and multivesicular bodies (MVBs). Subsequently, the MVBs either fuse with lysosomes or the plasma membrane. Upon fusion of the MVB with the plasma membrane, the vesicles are secreted into the extracellular milieu to become exosomes [22,23,24]. The literature highlights that the formation of intraluminal vesicles in MVBs is determined by at least four partially independent processes directed by the endosomal sorting complexes required for the transport class of proteins [25], the tetraspanin positive for the classification determinant (CD)63 marker [26], specific glycan modifications, and/or lipid-dependent mechanisms [27,28,29]. Other studies have found that proteins such as Ras-associated binding proteins (Rab)11, 27, and 35 have roles in exosome exocytosis and secretion in at least some cell types [30,31,32]. The theories on the generalized and specialized Rab-dependent intracellular trafficking mechanisms for exosomes’ secretion may explain the differences in the exosomes’ content and functions or may provide insights into the mechanisms by which exosome sub-populations are produced [33,34,35]. However, the presence of multiple mechanisms has confounded attempts to define the fundamental biology underlying exosomes’ formation and cargo sorting into exosomes [36]. It is also recognized that exosomes are produced by most cells of the body and can be recovered from bodily fluids [5], such as the prostasomes, formed in endosomes of prostate epithelial cells and therewith secreted into semen [37,38]. Therefore, further research will consolidate the mechanisms still in question and should provide more evidence to explain how exosomes influence the cellular functions within physiological and pathological ranges.

### 2.2. Kinetics and Mechanism of Action (MOA)

Since exosomes are currently regarded as distinct cellular entities, their specific roles and their ways of interacting with various cells at various locations have also been researched intensively. Their kinetics have also been scrutinized, and studies have concluded that it is more difficult to estimate the half-life of human exosomes in blood than it is to estimate exosome half-lives in lab animals. In athymic nude mice, the half-life of extracellular vesicles in vivo has been estimated at 30 min and the clearance 6 h after an intravenous injection [39], whereas, in humans, estimation has relied on complex calculations of hemodynamics and the half-life has not been directly measured. Moreover, the variety of cargo to be shared between cells (proteins, deoxyribonucleic acid (DNA), messenger ribonucleic acid (mRNA), and non-coding RNAs) [8,9,40] indicated that, from the mechanism of action (MOA) perspective, exosomes ensure intercellular communication and target various locations such as reticulocytes during their maturation [8,41], sperm during fertilization [42,43], immune cells during pregnancy [6], or the central nervous system (CNS) [44]. It was further considered that such interaction was leading to the exchange of genetic information and reprogramming of the recipient cells [45]. Apart from the targets involved in physiological processes, exosomes are essential to many lethal elements of cancer, inflammation, and other pathophysiological conditions. Studies have revealed the impact of exosomes on cancer-related processes, specifically their active interference with the tumor microenvironment [46], involving many cell types within the tumor [47]. Exosomes also accumulate at locations in target tissue where primary metastases appear first [46], and their production of extracellular adenosine triphosphate (ATP) could create a local inflammatory site where the metastasizing cell could settle down and create pre-metastatic niches [48]. The involvement of exosomes in malignant and inflammatory processes is noteworthy. Exosomes derived from gastric [49] or breast cancer [50] cells stimulate the activation of the nuclear factor kappa-light-chain-enhancer of activated B cell (NF-kB) pathways in macrophages. The consequence is an increased level of pro-inflammatory factors which, in turn, promote tumor cell proliferation and migration.

The excessive number of exosomes secreted by tumors may transfer messages from tumor cells to immune cells and stromal cells [51]. It was demonstrated that tumor-derived microvesicles induce, expand, and up-regulate biological activities of human regulatory T cells (Treg) [52]. Moreover, tumor-derived exosomes (TEXs) directly downregulate signals by the T cell receptors and other functions [53]. It was ultimately established that TEXs were more potent than mesenchymal stem cell-derived exosomes to induce regulatory responses by upregulating the production of FOXP3 (a protein involved in immune system responses), interleukin (IL)-10, and Transforming Growth Factor β (TGF-β – a polypeptide that regulates multiple biological processes) [54]. 

Exosomes can mediate the CNS neuronal and glia communication in pathological conditions such as glioblastoma via the transported RNA and proteins that promote tumor growth [55] or via a tumor suppression signaling network directed by miR-1 [56]. 

The capability of exosomes to carry inflammatory mediators, either proteins or miRNA, makes exosomes key role-players in systemic and local inflammatory pathways and implicitly in the pathogenesis of high incidence diseases such as cancer, diabetes mellitus type 2, rheumatoid arthritis (RA), inflammatory bowel diseases, or degenerative diseases [57]. It has been hypothesized that the RA exosomes are related to the induction of an osteoarthritis-like phenotype in both in vitro and ex vivo models. Further evidence is provided by exosomes that originate from synovial fibroblasts, which carry a membrane-bound form of TNF-α, allowing their uptake by anti-CD3-activated T cells and protein kinase B (AKT) and the activation of NF-kB. Moreover, activated T cells become resistant to apoptosis [58]. According to a recent hypothesis, exosomes are involved in chronic inflammation which describes the obesity disorder: exosomes from adipose tissue of obese subjects contained an altered level of 55 miRNAs, which mainly regulate TGF-β and Wnt/β-catenin signaling key cellular functions, including anticancer modulation [59,60]. Zhao et al. suggested that cross-talk between exosomes from Adipose-Derived Stem Cells (ADSC) and macrophages facilitated insulin sensitivity based on immune and metabolic homeostasis in white adipose tissue via anti-inflammatory M2 phenotype (alternatively activated macrophages) pathways [61]. Furthermore, exosome-secreted proteins interfere with effective cancer drug delivery due to stimulated fibroblast growth resulting in a desmoplastic reaction [51], or when they transfer chemo-resistance properties to nearby cells [62]. 

Since exosomal components are cell type-specific, stable, and accessible from body fluids, and they change according to the physio-pathological state of the cells of origin, they represent new research and clinical targets for diagnosis and therapy. However, fast, high-throughput, and reproducible methods for exosome isolation and molecular analysis are necessary for clinical implementation as a biomarker for liquid biopsy [36]. 

## 3. Challenges in Exosome-Based Research

### 3.1. Heterogeneous Morphology of Exosomes

It has been recorded that exosomes are vesicles with various densities ranging from 1.13 g·cm^−3^ to 1.19 g·cm^−3^ [6,63] and sizes from 30 nm to 200 nm [64,65]. The measurement techniques used include transmission electron microscopy (TEM), cryo-electron microscopy (cryo-EM), atomic force microscopy (AFM), dynamic light scattering (DLS) analysis, and flow cytometry (FC). Besides the size of the nanovesicles, other morphological details have significantly contributed to a better understanding of exosomes as complex entities [63,66]. A lipid bilayer membrane protects, and other homeostatic mechanisms help avoid, the aggregation of packed material (proteins, RNA, DNA) [65,67]. This lipid bilayer membrane expresses the same orientation of the parental cell plasma membrane and reflects the composition of the multi-vesicle endosomes (MVE) [11]. The exosomal membrane is enriched and substances such as flotillin, annexins, GTPases (guanosine triphosphate hydrolase enzymes), ceramides, cholesterol, sphingomyelin, endosome-specific tetraspanins (positive for the markers CD9, CD63, CD81, CD82), MVE biogenesis-related proteins (Alix, TSG101), or major histocompatibility complex of class (MHC)-II were identified [7]. These membrane markers are considered to be characteristic of the cell of origin, even though the content of the exosomes mimics, to some extent, the cell of origin [6]. For instance, studies reflected that antigens expressed on exosomes’ surface can be used for their selective isolation, like in the case of CD105 for endothelial cell exosomes (EC-EXs) and CD34 for endothelial progenitor cell exosomes (EPC-EXs) [68]. Moreover, in order for exosomes to fulfill their tasks and adapt to shifting conditions, they most likely bring a tool kit of essential and fluid-specific active surface enzymes [69]. 

The exosomal cytosol comprises a significant number of diverse biomolecules: lipids, specific proteins, and nucleic acids [70]. Vesicleopedia and ExoCarta included more than 14,000 biomolecules reported over time and mentioned an impressive number of proteins, mRNAs, and micro-RNA (miRNA or miR) in the exosomes from different species and tissues [71]. The cargo content can change according to each cell type, even though it has some proteins (transmembrane and cytosol proteins) in common in exosomes from a variety of sources [10] or it excludes many of the most abundant cellular proteins [72]. For example, the chaperone, HSP 70 (70kD heat shock protein), and tetraspan proteins are common to various exosomes, and it has been stipulated that only proteins that are not attached to the cytoskeleton are targeted for externalization [73]. Furthermore, glycophosphatidylinositol (GPI)-anchored proteins like GPI-anchored acetylcholine esterase and GPI-anchored prions are eliminated via exosomes and infectious PrPsc (a misfolded form of the cellular prion protein) and the native prion protein (PrP) are secreted via exosomes [74]. 

The transferrin receptor is a specific protein of the newly released circulating reticulocyte and the major protein in the exosomes derived from red blood cells (RBC). However, little or no transferrin receptors were found in exosomes derived from lymphocytes and dendritic cells, which instead showed the major histocompatibility complex of class (MHC) I and II molecules as a major fraction of their protein cargo. The exosomes released by the enterocytes are another example. The cytosol of these exosomes varies in cargo according to the location of the MVBs prior to their fusion with the plasma membrane of the enterocytes. The exosomes released from the MVBs at the basolateral surface of the enterocytes contained peptides, including the MHC I and II peptide complex, while the MVBs reported only in the apical compartments of the intestinal cells and rich in human leukocytes antigen DM (HLA-DM) were associated with no exosome release [75]. One more example of specific cargo is one of the cells in the renal tubules. The exosomes containing aquaporin and released into urine were considered the first ones excreted from the body with a recognizable and characteristic protein [69]. Furthermore, epididysomes were identified as exosome-like vesicles rich in sphingomyelin released by epididymis. Their essential role was associated with the transport of protein content towards the maturating spermatozoa via the long passage through the epididymis. Another example of an exosomal protein, despite being inconclusive, is lactadherin, one prominent peptide of dendritic cells [76]. However, there are currently no known exclusive protein markers for exosomes and the controlling mechanisms of the sorting of these different molecules into exosomes [77,78].

Concomitantly, studies have evidenced a wide range of small exosomal non-coding, regulatory micro RNA (miRNA) molecules, functional messenger RNAs (mRNA), exosomal shuttle RNA (esDNA), genomic DNA, and single-stranded DNA (ssDNA) [40]. Similar to the protein content, the miRNA and mRNA content of exosomes was noted to be a partial reflection of the cells of origin [40,79]. 

Furthermore, the lipidomic profile of exosomes indicated that their lipid cargo is closer to one of the MVBs rather than corresponding to the originating cells [80].

Besides the cell type-based variability of the molecular content of exosomes, there is one factor which significantly relates the cytosol changes to the physiological or pathological conditions of the cells. Importantly, such changes can be substantially different from the cell of origin, indicating a highly controlled sorting process [81]. 

Double-stranded DNA present in exosomes from cancer cells expressed the specific mutational status of the originated cells [82], and the protein composition of the tumor cell-derived exosomes can indicate the cell of origin after their characterization. The essential hypothesis is that, morphologically, exosomes originating from cancer cells are considered to be reservoirs of cancer biomarkers [62], while exosomes containing the prion protein, β-amyloid (Aβ), and α-synuclein, as neurodegenerative disease-associated proteins, are considered markers for those medical conditions [83]. Palmitoylated and tetraspanin proteins present on the surface of exosomes secreted by breast cancer cells, as well as miRNAs (miR-195, miR-21, miR-484/191) and some proteins (CD9, CD63, CD81, CD24, p-selectin, survivin and its splice variants,) are differentially expressed in certain stages and types of breast cancer (BC) in the extracted exosomes. 

It is also interesting that studies on mice showed a correlation between exosomes, obesity, and insulin resistance: the miRNAs isolated from the obese mice fat tissue macrophages triggered glucose intolerance and insulin resistance in non-obese mice [84]. Moreover, the exosomes isolated from synovial fluid with the presence of citrullinated proteins [85] are further involved in RA etiology. Also noteworthy is the frequent appearance of glycolytic enzymes in proteomics on exosomes and prostasomes and their possible relationship with lipid rafts-enzymes in the glycolytic pathway [71,86].

It was also hypothesized that the exosomes derived from normal cells may express the cells’ therapeutic potential, while the exosomes from diseased tissues may be important for the maintenance and spread of pathological processes. Therefore, the potential of a defined morphological profile will highlight the progress of the studied diseases and a clear morphological map is of great importance in identifying and isolating the exosomes for either diagnosis or monitoring of disease progress and therapy. 

### 3.2. Liquid Biopsy

A liquid biopsy is a medical tool that can be used for various diagnostic applications to accompany diagnostic protocols, molecular monitoring, or staging of diseases, especially cancer. The peripheral blood is the most common biofluid containing cells and/or fragments of pathologically modified cells and allows liquid biopsy diagnosis and monitoring upon analyzing the biologic materials. This type of analysis is considered a potent instrument despite the arguments regarding the extent of its accuracy due to a lack of high sensibility and specificity. For instance, a liquid biopsy may provide a focused screening among individuals at risk of various diseases, ranging from cancer to inflammatory and degenerative ones, and consecutively reduce the medical costs. To date, however, the only approved indication for the use of a liquid biopsy in cancer diagnosis is the use of blood for the detection of required activating mutations (geftinib) or the inhibiting T790M mutation in the epidermal growth factor receptor (EGFR) gene (osimertinib), if no adequate quantities of tumor tissue are available. Furthermore, the use of an exosomes-based liquid biopsy as dynamic monitoring is also one approach in cancer therapy. However, there are considerations triggered by extensive research on the biology of circulating tumor cells (CTCs), circulating tumor DNA (ctDNA), and exosomes regarding their clinical applications. If the challenge in CTCs and ctDNA is related to the capability of detecting single mutations applied to early detection and residual disease, in the case of exosomes, the challenge resides in the overlap of surface protein markers and associated heterogeneity among exosomes derived from multiple cell types in physiological or pathological states. Therefore, there is a stringent requirement for better exosomal markers for their high-purity isolation [87,88]. 

### 3.3. Technology for Isolation and Enrichment 

Numerous studies have explored the diagnostic and therapeutic potential of exosomes and evidenced many conventional methods that involve a direct analysis of cellular materials from biopsy samples. In the case of a fluid biopsy for personalized medicine, a key challenge is the lack of efficient and standard techniques for isolation and downstream analysis. Current conventional isolation methods, such as ultracentrifugation, precipitation, filtration, chromatography, and immune-affinity-based approaches, rely on size differences or on specific surface biomarkers. Moreover, the development of microfluidics is particularly significant as liquid biopsy platforms employ a range of isolation approaches, such as immunoaffinity, membrane-based filtration, nanowire trapping, acoustic nanofiltration, deterministic lateral displacement, and viscoelastic flow sorting. However, all the previous works stressed the need for established adequate scalability, standardization, and validation of the isolation and downstream proteomic and sequencing analysis approaches [89]. These aspects will be further addressed in Section 4.

## 4. Microfluidic-Based Approaches for Exosome Investigation in the Laboratory

### 4.1. Exosome Isolation 

**Figure 1:**
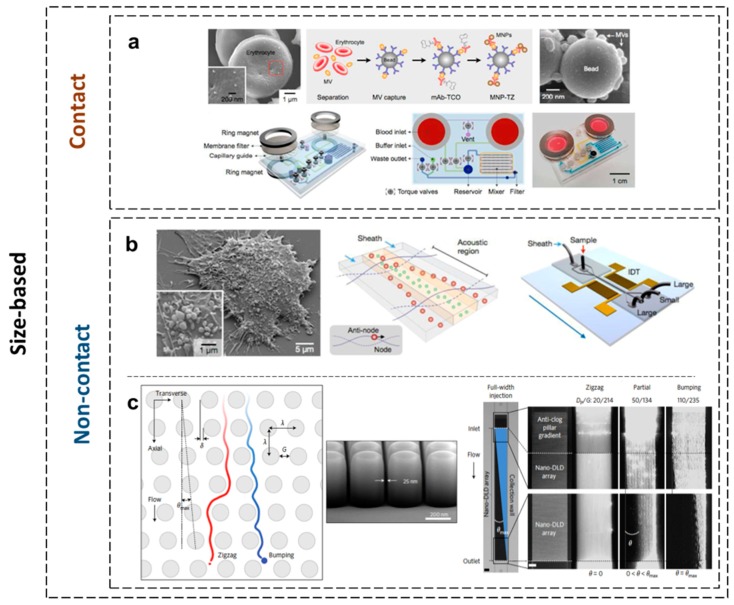
Size-based exosome separation techniques. (**a**) The nano-porous physical filtering technique. The microvesicles (MVs) are separated from blood and captured by antibody-coated microbeads with a mean diameter of 1 μm. Immunomagnetic labeling subsequently renders the beads superparamagnetic [95] [reproduced with permission from [95], © 2013 American Chemical Society (ACS)]. (**b**) The non-contact acoustic nanofiltering technique where the MVs are moved in different locations of the microfluidic channels according to their acoustic response. Interdigitated transducer (IDT) electrodes shown in the schematic are used to generate surface acoustic waves across the flow direction [96] (reproduced with permission from [96], © 2015 ACS). (**c**) The nano-deterministic lateral displacement (nano-DLD) non-contact technique. Nanoparticle sorting accomplished using pillar array chips and the separation of polystyrene beads using a hydrodynamically focused jet in the nano-DLD array [97] (reproduced with permission from [97], © 2016 Nature publishing group).

The essential advantage of exosome analysis over blood-based markers such as CTCs is the access to a larger population of biomarkers [90]. As exosomes represent an enriched source of biomolecules, including proteins and nucleic acids, they are hypothetically more reliable peripheral biomarkers for malignancies and neurodegenerative, cardiovascular, or other diseases than neat cerebrospinal fluid (CSF), blood, or urine sample analysis [91]. It is generally accepted that the protocols for the isolation and analysis of microvesicles from blood are laborious [92,93]. The conventional assays are time-consuming, and they exhibit a low recovery factor. They also require ultracentrifugation to concentrate large volumes of samples with extensive processing for subsequent detection, such as Western blot (WB) and enzyme-linked immunosorbent assays (ELISA) [94]. Since the current methods are difficult to implement in clinical settings, especially in studies that involve a large throughput or rare molecular targets, new technologies have emerged as a series of miniaturized systems. Microfluidic technology has been adapted to address both isolation and analysis. The currently used microfluidics-based exosome separation methods are classified into three categories: size-based, immune-affinity-based, and dynamic categories. Size-based exosome separation devices include nanofilters, nanoporous membranes, or nanoarrays to trap the vesicles when fluids flow through the channel. For instance, one sandwich-like device comprised one detachable membrane filter (with 0.1 μm pores), two permanent ring magnets to size-selectively enrich exosomes from large sample volumes [95], and one microfluidic circuit inserted beneath the membrane to guide the filtered exosomes to the collection channel (a schematic drawing is illustrated in Figure 1a). The design enabled the easy replacement of filter units when processing large sample volumes. The microfluidic setup was fabricated using soft lithography and consisted of three polydimethylsiloxane (PDMS) layers bonded on a glass slide. The driving of the plasma inside the microfluidic channels was performed through negative pressure using mechanically actuated valves (fabricated in the PDMS structure). The PDMS layers were assembled via adhesive bonding using uncured PDMS, while the PDMS structure was irreversibly bonded to glass using classical O_2_ plasma activation. The microfluidic system directly isolated microvesicles (MVs) from the blood before labeling them with magnetic nanoparticles analyzed by a miniaturized micro-nuclear magnetic resonance system (µNMR). The high-sensitivity on-chip analysis of small volumes of blood (<200 µL) measured the concentration of targeted red blood cells (RBC)-derived MVs positive for CD235, CD44, CD47, and CD55 markers. Despite the low specificity, the platform enabled the efficient isolation of nanoscale MVs directly from stored blood units and for point-of-care testing (POCT) of blood products. 

An acoustic nanofilter system was demonstrated to separate exosomes in a contact-free continuous flow manner from small samples. A schematic drawing of the microfluidic chip is presented in Figure 1b. It used surface acoustic waves (SAW) in order to separate the extracellular vesicles according to their size and density. The important aspect of the device is that the separation occurs under continuous flow (having a reduced risk of clogging). The SAW generates a drag force dependent on the volume of the particle. Other parameters that can be operated in order to control the particle movement are the acoustic power and the velocity of the fluid. The device presents two inlets: one for the sample and one for a buffer. The sample is focused in the middle of the channel, while a pair of interdigitated electrodes (IDE) generate the acoustic waves perpendicular to the microfluidic channel. The small nanovesicles are focused in the middle of the channel and collected on a central outlet, while the large size particles are deflected to the side outlets. The IDE are patterned on a piezoelectric substrate (LiNbO_3_), while the microfluidic structure is fabricated using classical soft-lithography. It produces a high separation resolution and yield. The results also evidenced its potential as a fast and effective exosome isolation method [96]. 

Later on, the method employing the nano-deterministic lateral displacement (DLD) arrays built within a microchannel demonstrated the high-resolution separation of nanoparticles of diameters from 20 nm to 110 nm, inclusive of exosomes. [97]. The nano-DLD method (coined by Huang et al. [98]) consists of flowing the sample through a nanopillar array (Figure 1c). The pillars are in rows with an established pitch, gap, and diameter. The critical aspect of the design is the row-to-row shift responsible for the nanofiltration process. If the diameter of the particle is larger than the critical diameter, the nanosized particle will migrate laterally with an angle defined by the geometry of the nanopattern (pillar pitch and row-to-row shift). If the diameter of the particle is smaller than the critical diameter, there will be no lateral migration of the particles. The method showed potential for on-chip sorting and quantification of the exosomes and other extracellular vehicles (EVs). The work also proved the possible fabrication of a nano-pillars array with a 25 nm gap. The nano-pillars were patterned in silicon by an optimized deep RIE process through an SiO_2_ mask. The aspect ratio (depth/gap) was 10:1. The microfluidic structure was closed by a glass wafer. Special attention was given to the surface modification of the microfluidic channel in order to avoid trapping (clogging) of the exosomes. The experimental results proved that the displacement of 20 nm nanoparticles can be achieved with a gap of 42 nm. The tested device showed that a polydispersed population of exosomes can be grouped in terms of the size range. The measurement of the uniformity and size was performed using scanning electron microscopy (SEM).

**Figure 2:**
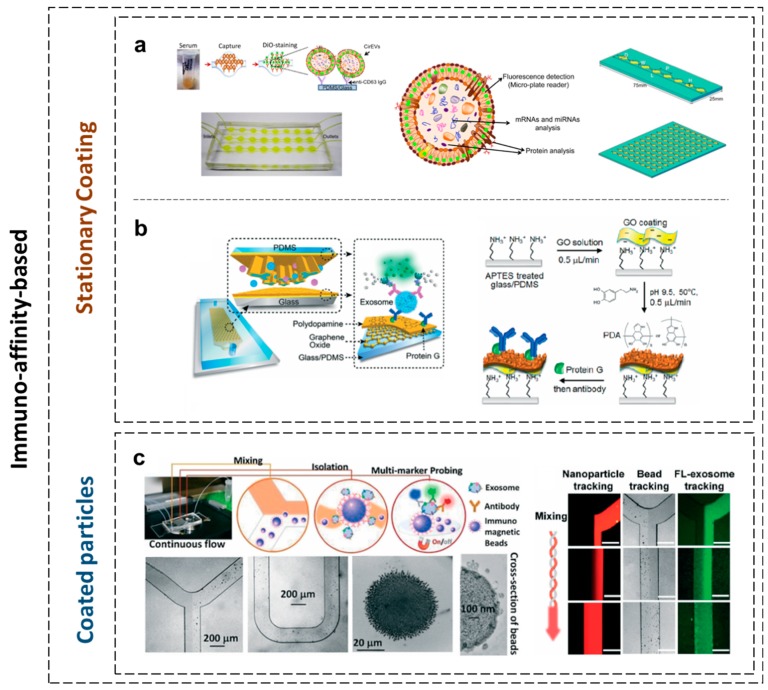
Immuno-affinity-based exosome separation. (**a**) The ExoChip and its operation procedure used for exosomes’ isolation and analysis, consisting of eight equally spaced circular chambers connected through a wide channel, allowing its use for analyzing multiple samples simultaneously. The polydimethylsiloxane (PDMS)-based working prototype ExoChip (with three channels) depicts the flow of serum for exosomes’ capture in a typical experimental setup [99], [reproduced with permission from [99], © 2014 Royal Society of Chemistry (RSC)]. (**b**) The nano-interfaced microfluidic exosome platform (Nano-IMEX). Schematic of a single-channel PDMS/glass device, with the exploded-view highlighting the coated PDMS chip containing an array of Y-shaped micro-posts. The surface of the channel and micro-posts are coated with graphene oxide and polydopamine as a nanostructured interface for the sandwich enzyme-linked immunosorbent assays (ELISA) of exosomes with enzymatic fluorescence signal amplification [100] (reproduced with permission from [100], © 2016 RSC). (**c**) The ExoSearch platform. A robust, continuous-flow platform providing the enriched preparation of blood plasma exosomes for in situ, multiplexed detection using immunomagnetic beads for the quantitative isolation and release of exosomes [101] (reproduced with permission from [101], © 2016 RSC).

Furthermore, an immunoaffinity-based microfluidic device achieved specific separation since it relied on specific biomarkers on the exosomes’ membranes. These devices comprised modified microchannels with antibodies or magnetic beads with adapted affinity. One example shown in Figure 2a is the ExoChip [99]. The microfluidic system consists of one or multiple channels, where each channel presents multiple wells that are connected through a narrow channel. In the wells, the velocity of the fluid is reduced (due to the dimension of the chamber) to allow the interaction between exosomes and the surface of the microfluidic structure (functionalized with antibodies against CD63 – an antigen overexpressed in exosomes). The connecting channel between wells allows mixing of the sample due to the gradient of the velocity. The microfluidic device was built using soft lithography (fabricated in polydimethylsiloxane-PDMS and bonded on glass using plasma treatment). This device enabled the profiling and quantifying of exosomes when subsequent specific staining with a fluorescent carbocyanine dye (DiO), immuno-electron-microscopy, and WB of the recovered exosomes with intact RNA were applied. Similar solutions have been developed to facilitate rapid, accurate, highly sensitive screening and high specificity diagnostic tests. An example depicted in Figure 2b is the nano-interfaced microfluidic exosome platform (Nano-IMEX) presented in [100]. The device consists of a microfluidic channel processed in PDMS using soft lithography and bonded in a glass slide. The mixing process is assured by PDMS pillars. The surface modification was performed on a chip with graphene oxide and polydopamine (a biocompatible material), while controlling the flowrate and the temperature of the substrate. The device was tested on 2 μL clinical plasma samples from patients with ovarian cancer and healthy patients.

The newly emerged devices were designed as methods to collect intact exosomes directly from biological samples and replace ultracentrifugation or other precipitation methods. ExoSearch (Figure 2c) was also developed as a robust design for in-situ detection and analysis [101]. As depicted in Figure 2c, magnetic beads and the plasma sample containing exosomes are injected in a “Y”-shape microfluidic channel and mixed using a classical serpentine (for improved beads-exosome capture). Magnetic beads with attached exosomes are magnetically trapped in a microchamber where a mixture of fluorescent-label antibodies stained the nano-vesicles. The chip was tested for blood-based ovarian cancer diagnostics. The chip was fabricated using PDMS technology, with the magnet embedded in the PDMS structure.

**Figure 3:**
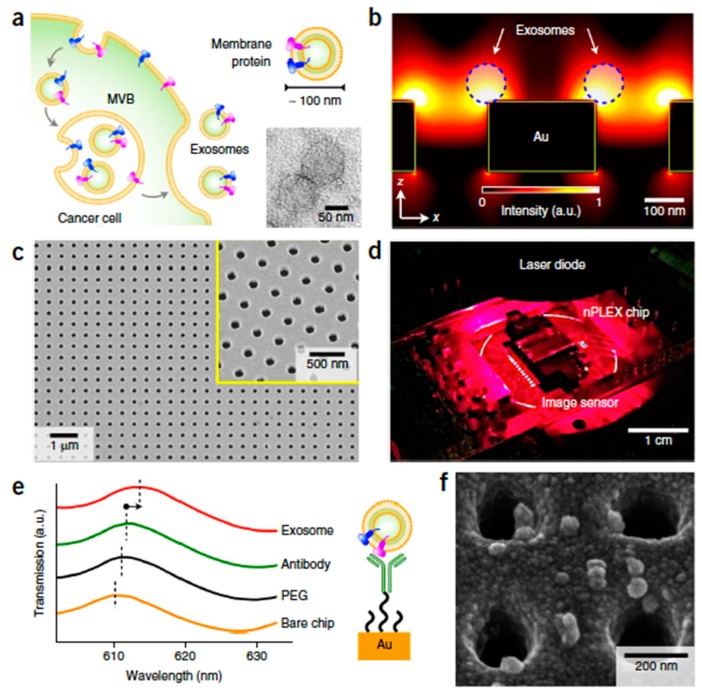
Detection of exosomes using the nano-plasmonic exosome (nPLEX)—sensor developed by Im et al. [110]. (**a**) Exosomes secretion. (**b**) Simulation results of the electromagnetic field justifying the trapping of exosomes on the sensor’s surface. (**c**) SEM image of the nanoholes. (**d**) nPLEX chip and the imaging system. (**e**) Transmission spectra for exosome detection with nPLEX. (**f**) SEM of the nanoholes with trapped exosomes (reproduced with permission from [110], © 2014 Nature publishing group).

The microfluidic devices which use microscale channels to manipulate small liquid samples and carry reactions in parallel to offer tremendous opportunities were coupled with the emerging multi-dimensional nanostructures. The application of multidimensional nanostructures, including nanoparticles [102,103], nanopillars, nanowires [104], nanoporous layers, and graphene-based materials [105,106,107] when integrated with microfluidic channels in molecular and cellular separation, facilitates biological separation in microfluidic channels and provides insights into the future of nanostructure-integrated microfluidic platforms and their role in biological and biomedical applications [95,96,99,102,108,109,110,111,112,113]. Im et al. proposed the plasmonic exosome-sensor (PLEX) sensor for exosomes detection and used surface plasmon resonance [110] with potential application in diagnostics, as shown in Figure 3. In their design, a nanohole array was functionalized for trapping exosomes originating from ovarian cancer cells. Wang et al. [114] reported a ciliated nanowire-on-micropillar structure to isolate exosomes using only conventional microfabrication techniques. The micro-scale fluid channels allowed the efficient processing of a small sample with a volume of 100 µL at a low risk of clogging. Micrometer-scale nuclear magnetic resonance (μNMR) was also employed for the magnetic detection and analysis of exosomes with the help of this type of microfluidic device [102]. Petersen et al. demonstrated the efficacy of field flow fractionation methods in separating exosome subpopulations without immune-affinity or other labeling steps [115].

Moreover, the nanoplasmonic exosome detector in which surface plasmon resonance (SPR) is used for label-free exosome sensing, is added to the existing miniaturized systems for sample preparation (microfluidics) and for protein analyses (analytical tools) [116]. The immune-magnetic exosome RNA (i-MER) platform proposed an on-chip method for exosome isolation, followed by RNA extraction, reverse transcription, and real-time amplification. The isolation of the exosomes from serum was possible with the use of magnetic microbeads holding affinity ligands (anti-CD63, anti-EGFR). The exosome population was then lysed, and the RNA was transferred on glass beads for further steps: reverse transcription, RT PCR, and quantification [111]. Recently, a surface-enhanced Raman scattering (SERS)-based detection method for tumor-derived exosomes was described for both qualitative and quantitative detection. SERS nanoprobes and magnetic nanobeads can capture the exosomes by forming a sandwich-type immuno-complex, which can be precipitated with a magnet, and thus SERS signals can be detected in the precipitates whenever target exosomes are present [117]. Surface-enhanced Raman Spectroscopy (SERS) was used for the evaluation of exosomes enriched from normal and pancreatic cancer cell lines. The principal component differential function analysis (PC-DFA) method determined the tumor-specific spectrum. The differentiation accuracy was estimated at 90%. This method exhibited up to an 87% and 90% predictive accuracy for healthy controls (HC) and early pancreatic cancer (EPC) individual samples, respectively [118].

In comparison, Chen et al. [93] presented another easy and rapid method to isolate exosomes using the immunoaffinity principle. Binding antibodies onto the chip surface could then be used to capture exosomes. This is a faster and simpler method of exosome isolation compared with current protocols since it involves high-speed centrifugation and filtration.

Unfortunately, the immunoaffinity-based separation microfluidic devices can only separate exosomes that have highly represented antigens (targeted proteins of interest) on their surfaces. Therefore, dynamic methods were developed to integrate external forces such as ultrasound or flow field-flow fractionation (FIFFF) into new microfluidic technologies for the faster and easier separation of exosomes. For instance, Davies et al. [119] avoided the usage of antibody selection and developed a microfluidic filtration system to isolate exosomes and derive mRNA from whole blood samples. Their method avoids laborious centrifugation and antibody-based affinity purification. By integrating a porous polymer monolithic membrane (PPM) into a poly(methyl methacrylate) (PMMA) chip, vesicles could be separated from cells and debris according to their sizes. By adjusting the size of the membrane pores, they were able to selectively isolate exosomes and reject cellular components. Moreover, another acoustic nanofilter system separated extravesicles (EVs) by density and size with an 80% recovery rate for exosomes [96]. However, the data on the FIFFF device presented the noncomplicated nanostructure and not the purity and recovery rate of the separated subcellular species in the studied samples [120]. The most commonly used techniques to purify exosomes and their related clinical potential are briefly presented in Table A1.

Extensive work has addressed the clinical potential of the conventional and newly emerged microfluidic-based devices [15,99,121]. In contrast with the detection deficiencies of *CellSearch***^®^**, exosomes have been readily detectable in early- and late-stage pancreatic cancer, melanoma, glioblastoma, and other cancers for which CTC isolation is currently difficult or impossible [122,123,124]. Furthermore, integrating microfluidic devices, biosensors, and a smartphone for liquid biopsies at the POCT has become a reality. Lab-on-a-chip systems can be used for the isolation of tumor-derived components present on various biofluid samples, such as peripheral blood, urine, or saliva. Moreover, smartphones have started to be used for optical detection and data analyses for POCT and personalized therapeutic schemes. 

Despite the advances of new technologies [125], there is a particular need for a standardized performance of the existing models towards a higher reproducibility of the exosomes’ recovery despite the variations within samples. 

### 4.2. Exosome Analysis and Characterization

Once isolated, exosomes which usually exist in large amounts in biological fluids have to be analyzed from banked and frozen biological samples [126] for specific markers which characterize the primary tumor, the pathological process, and the tumoral microenvironment [127]. The morphological and functional aspects were considered for the analysis of exosomes. Many methods were employed, such as WB, a nanoplasmonic colorimetric assay, atomic force microscopy (AFM), scanning helium ion microscopy (HIM), qNano, transmission electron microscopy (TEM), an immunofluorescence (IF) assay, and a reverse transcription real-time polymerase chain reaction (RT-PCR). Each of these methods can contribute information regarding the particle size and counts, molecular profiles of the transmembrane proteins, and the cargo and functional aspects of the exosomes. WB results, for instance, indicated the exosomes’ ability to modify functions of immune cells (NK cells or T cells), or a lateral flow immunoassay (LFIA).

The phenotyping of exosomes identified disease-specific protein targets for solid tumors such as: the epithelial cellular adhesion molecule (EpCAM) for colorectal [128,129] and ovarian cancers [110,130], epidermal growth factor receptor (EGFR) for brain cancers [127] and human epidermal growth factor receptor 2 receptor (HER2/neu) for breast cancer [131], the membrane protein insulin-like growth factor-1 receptor (IGF-1R) and intravesicular phosphorylated-IGF-1R (p-IGF-1R) directly in the plasma of early-stage non-small-cell lung carcinoma (NSCLC) patients [109], cytokeratin (CK)19 for colorectal cancer (CRC) cells, a tumor-associated glycoprotein (TAG)72 for five fluorouracil (5-FU)-resistant CRC cells, and a carbohydrate antigen (CA)125 in highly metastatic CRC cells [132].

Studies revealed that in addition to targeted proteins, exosomes also contain nucleic acids: RNAs [55,133] and DNAs [134]. Subsequent studies demonstrated the utility of exosomal DNAs for tumor molecular analysis, presenting them as reflective tumor markers [134,135]. Moreover, the various types of RNAs can also be used as reflective disease markers, since their levels or patterns showed a correlation with various types of cancer:

- exosomal miRNA in lung adenocarcinoma [136,137,138], pancreatic cancer [139], glioblastoma [93], melanoma [119],
- mRNA mutants (EGFRvIIImRNA) and miRNAs in glioblastomas [133], ovarian cancer [68], prostate cancer [140,141,142], and gastric cancer [143].

It has been noticed that alterations in mRNA expression levels clearly have functional consequences of diagnostic importance, so the exosome shuttle RNA (esRNA) comprising mRNA and miRNA is foreseen as a biomarker for early cancer diagnosis and surveillance [133]. Moreover, exosomal cell-free circulating RNA (cfRNA) is possible to detect in the blood since it is stable [144,145].

Since most enrichment methods are employed for exosome isolation, and single marker targeted enrichment and detection of tumor-derived exosomes in the circulation of cancer patients were insufficient for separation due to the lack of specificity, the detection of DNA mutations is required for sensitive and specific analysis. For instance, in pancreatic cancer, a physical-cum-biological-based targeted enrichment method combined with mutant Kirsten rat sarcoma viral oncogene homolog (KRAS) detection using droplet digital PCR increased the sensitivity from 44% to 73% by reducing the background signals, despite the relatively low accuracy [146]. Several attempts recorded the successful identification of GPC1 (Glypican 1—a protein expressed on the surface of pancreatic cancer-derived exosomes) at an absolute sensitivity and specificity. One approach combined differential centrifugation enrichment followed by flow-cytometry [122], while a fluorescence *in situ* hybridization (FISH) approach detected GPC1 mRNA directly from the serum of patients with pancreatic cancer without the need for exosome purification and with potential as an accurate bio-chip platform [147]. In the quest for such accurate bio-chip platforms accompanied by promising results in the discrimination of patients with pancreatic cancer from healthy controls and benign patients with a full accuracy, Lewis et al. proposed and reported one microarray chip capable of the dielectrophoretic isolation of exosomes from whole blood and in situ detection of GPC1 using immunostaining [148]. However, the best combination appears to be achieved when an miRNA panel and a surface protein panel are considered [149].

Since the coding and noncoding exosomal RNA profiles proved to be of diagnostic importance, the analytical methods play crucial roles in this process. Paolini et al. [150] recently approached the currently available separation and analytical methods and evaluated the impact of their combinations upon the purity and further biophysical and biochemical analysis of the samples, including the impact of the residual matrix upon the biological activity of the exosomes isolated through purification. They presented the biochemical characterization of exosome preparations from a 1 mL multiple myeloma (MM) pool with different protocols for exosome preparation: differential centrifugation steps (P3), purification with a precipitation kit (Exo PK), and a discontinuous iodixanol gradient. The samples were electrophoresed and analyzed by WB for the presence of typical vesicular markers to analyze the influence of separation methods on analysis and biomarker-based diagnosis.

Another example is the nano-interfaced microfluidic exosome (nano-IMEX) chip based on a new graphene oxide/polydopamine (GO/PDA) nanocoating technology. It greatly enhanced the detection sensitivity and dynamic range, and enabled the quantitative detection of circulating exosomes directly from unprocessed plasma samples of a minimal volume of 2 μL, addressing one of the key challenges in the clinical development of exosomes as biomarkers. It also successfully distinguished ovarian cancer cases from healthy controls and quantified the expression of exosomal markers in a patient in response to cancer treatment [100]. The development of platforms which employed quantum dots (QDs) and fluorescence nanoparticle tracking analysis (NTA) also allowed phenotyping of the circulating epithelial tumor-derived exosomes [151,152].

**Figure 4:**
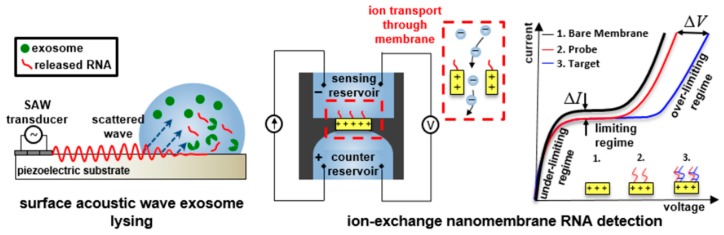
On-chip surface acoustic wave lysis and ion-exchange nanomembrane detection of exosomal RNA for the study and diagnosis of pancreatic cancer, developed by Taller et al. [139]. Reproduced with permission from the Royal Society of Chemistry (RSC).

Furthermore, several groups focused on POCT and monitoring platforms, which incorporate isolation coupled with a downstream analysis technique. In this direction, Lee et al. [96] showed label-free size-based purification of exosomes using an acoustic-based microfluidic device, while Taller et al. [139] designed a microfluidic chip for the analysis of exosomal miRNA in pancreatic cancer. They utilized two chips in conjunction (Figure 4): one using surface acoustic waves (SAW) for exosome lysis and another with an ion-exchange nanomembrane for RNA sensing. A transducer generates SAW, which refracts in the liquid sample and sets it in motion; at the same time, electromechanical coupling generates an electric wave. By targeting microRNA (miRNA), they extracted exosomal RNA from a pancreatic cancer cell line and achieved a 38% lysis rate, as well as a limit of detection of 2 pM. 

Chen et al. [93] presented an immunoaffinity-based microfluidic device that rapidly and specifically isolated exosomes from serum samples. They obtained microvesicles from small volumes of blood serum and cell culture condition medium to extract high-quality RNA. Furthermore, the Taylor group repurposed magnetic-activated cell sorting (MACS) to isolate exosomes from serum samples from early-stage ovarian cancer [140]. Vaidyanathan et al. [153] also developed an immunoaffinity-based highly specific device to isolate exosomes from an immunocapture site using nanoshearing (a tunable alternating current electrohydrodynamic-acEHD method). The exosomes captured on this chip were subsequently incubated with an anti-fluorescein horseradish peroxidase (HRP) antibody and tetramethylbenzidine (TMB) to induce colorimetric read-out for macroscopic identification. Dudani et al. [154] demonstrated the potential for the practical clinical use of the combination of the isolation of exosomes via inertial lift force in microfluidic channels and their quantification via immunocapture. The profiling of exosomes from glioblastoma multiforme (GBM) was investigated via μNMR. Blood samples of healthy and GBM patients were used in a pilot clinical trial. The results proved that exosomal protein profiling could permit accurate disease diagnosis and treatment surveillance [102]. Davies et al. [119] developed a microfluidic filtration system to isolate exosomes and derive mRNA from whole blood samples. They harnessed the electrophoretic mobility difference between soluble proteins and exosomes to increase the RNA extraction yield per unit of protein. The microfluidic technology greatly improved the limit of detection to 106 vesicles/mL, substantially reduced the sample consumption and analysis time, and enabled the probing of biological events that had previously been inaccessible [155].

We believe that the constant progress of microfluidic technologies for exosome isolation and molecular characterization with an emphasis on immediate clinical applications will ultimately lead to the development of a possible “gold standard” device and method which could be used to isolate, purify, and use exosomes as a valuable tool for the analysis of liquid biopsies. The minimal amount of preparation steps required for exosome enrichment with the direct use of serum or even whole blood [147] makes exosome analysis an ideal candidate for POCT personalized medicine. Since it has been demonstrated that a liquid biopsy targeting tumor-derived exosomes could be performed rapidly, coupling exosome-based liquid biopsy with small sample volumes [148,156] and a low cost would make the method applicable for real-time monitoring. 

## 5. Clinical Applications of Exosomes 

Integrated analysis of the overall exosome levels allowed the quantification of CD9 (+) and CD81(+) from human plasma in ovarian cancer (nano-IMEX) [100] and NSCLC [109], and CD63(+) in pancreatic cancer (ExoChip) [99]. The relevance of exosomes is highlighted in the review by Alberter et al., who indicated that in pancreatic ductal adenocarcinoma, the number of exosomes in the blood correlated with disease stage, and even early stages of the disease, were reliably detected with a 100% sensitivity and specificity [157]. Moreover, a large quantity of cancer exosomes have been detected in blood samples of patients with CNS tumors, located behind a partially intact blood-brain barrier, which typically do not release many CTCs [132]. The same work highlighted the isolation of exosomes from cell culture supernatants of colorectal cancer (CRC) cells, CRC interstitial fluid, and patients’ plasma-derived exosomes: CK19 was commonly expressed in exosomes derived from colorectal cells, TAG72 was mainly expressed in exosomes of 5-FU-resistant CRC cells, and CA125 in those of highly metastatic CRC cells. These provided a good prospect for cell exosomes as a novel, non-invasive clinical tool for diagnosing CRC and predicting its progression. Shao et al. published the results of a comparative protein analysis of circulating exosomes and highlighted a variety of disease-specific protein targets for solid tumors. Furthermore, it was observed that the exosomes’ specific types of RNAs and DNAs can be used as reflective disease markers, such as mRNA mutants (EGFRvIIImRNA) and miRNAs in glioblastoma; miRNA in lung cancer, prostate cancer, and gastric cancer; and DNAs for tumor molecular analyses [158]. µNMR enabled the quantification of specific exosome subpopulations, such as EGFR, EGFRvIII, PDPN, and IDH1 R132H, despite required off-chip isolation and lysis of the exosomes [15]. Apart from the morphological characterization, knowledge of the functional features of the tumor-derived exosomes (TEX) in terms of their interaction with immune and non-immune cells and the transfer of information to specifically suppress essential functions in the responder cells are considered a new frontier in hematological malignancies [159]. Liang et al. proposed a high-throughput platform for the filtration-based isolation and ELISA colorimetric-based detection of urinary bladder cancer-derived exosomes [160]. The clinical potential of nPLEX emerged from its high-level integration and multiplexing capacity as the label-free detection of ovarian cancer-derived EpCAM (+) and CD24 (+) relative to CD63(+) exosomes in ascites fluids [110]. Rapid inertial solution exchange (RInSE) proposed by Dunani et al. combined off-chip incubation steps with inertial focusing while isolating and capturing EpCAM(+)-labeled exosomes from pre-processed blood [154]. 

The concern about using blood-based biomarkers triggered studies on immunochemical methods that could be used to harvest and enrich brain-derived exosomes in blood in nonmalignant diseases. Such studies, using the neural cell adhesion molecule (NCAM) and L1 cell adhesion molecule (L1CAM), specifically analyzed levels of Aβ (amyloid beta) and p-tau (phosphorylated tau protein) from exosomes possibly derived from the brain in an attempt to design an exosome as a biomarker-based diagnosis tool for Alzheimer’s disease (AD) [67]. Other studies focused on the isolation of exosomes derived from SKNSH-SY5Y cells [161], CHO cells [162], N2a cells [163], mouse primary astrocytes and neurons [164], dendritic cells [165], and CSF [166] to evaluate their involvement in AD. Studies on the exosomes involved in Parkinson’s disease used various sources like SH-SY5Y cells [167], primary neurons [168], H4 cells [169], PC12 cells [170], or CSF in patients’ urine [171]. 

Besides having diagnostic value, the exosomes were identified as key players in the etiology of Rheumatoid arthritis (RA). Zhang et al. [70] introduced evidence in this direction and related the vesicles isolated from synovial fibroblasts to the induction of an osteoarthritis-like phenotype in in vitro and ex vivo models when treated with Interleukin-1β. 

Studies evidenced the sources of exosomes and their roles in other inflammatory-based diseases, such as inflammatory bowel diseases. The exosomes collected via an intestinal tissue biopsy and saliva were considered for the diagnosis due to an increased IL-8 level in human colonocytes [172], an elevated number of exosomes containing annexin-1 [173], or due to a marked difference in PSMA7 expression in the exosomes of inflammatory bowel disease patients compared to controls [174]. 

From the laboratory diagnostic procedure perspective, the fact that exosomes carry and protect diverse molecular information from cells’ degradation, primary tumors, and their microenvironment and that they can be analyzed from banked and frozen biological samples represent an avenue towards new miniaturized technologies [158]. Furthermore, a liquid biopsy can be employed as a source of mRNA and miRNA encapsulated in exosomes (exosome shuttle RNA) from biofluids such as the serum, urine, and saliva and can be used as the basis of on-chip genomics for early diagnosis and for the surveillance of various disorders [133]. 

## 6. Challenges and Perspectives

The current agreement that exosomes are stable and abundant in biofluids, as well as diverse, while specific in cargo, represents a stimulating trigger for their detailed research and the associated clinical implementation for diagnosis and disease monitoring. Moreover, the acknowledged profile of the state of art technique for their diagnosis and characterization, as well as of the unique set of challenges facing the clinical translation, open new avenues for research on ready-to-use all-in-one types of microfluidic devices. The profile of an ideal lab-on-chip tool has been contoured already and it is the focus of research: it is a miniaturized on-chip tool that is fast and highly sensitive and specific, has a high throughput, has constant accurate results upon repetitive use, and has a capacity for on-site analysis, thus being user-friendly, clinically reliable, and cost effective. Such a desiderate can be achieved if the developing instruments consider readily available biofluids uniquely handled as small samples. Moreover, the microfluidic platforms will support a combination of steps and methods from detection and isolation/enrichment to augmentation and final analysis of the exosomes. In the meantime, research needs to focus on the proposed platforms to offer several advantages in terms of simplicity of the protocols, smaller sample volumes, a lower concentration of exosomes required for analysis, higher throughput, and on-chip combined assays. Therefore, perfecting the isolation and analysis methods integrated by microfluidic platforms is crucial when considering the phenotyping or genotyping of exosomes from cell cultures or biological samples. The developed devices will complete the technical panel of the diagnostic laboratory for a qualitative and quantitative evaluation of exosomes. The ultimate goal of the microfluidic-based exosomal isolation and analysis is a successful clinical implementation and POCT applicable to a wide range of diseases, either benign or malignant.

Significant progress is still required to further address the perfect match between the type of biofluid collected and the desired lab application. For instance, exosomes could be beneficial for drug resistance monitoring. Future development of all the reviewed instruments and methods essentially targets their ability to isolate biomarkers from very small samples of biofluids, from samples which comprise very small amounts of biomarker(s), or from samples collected from cancer patients who express rare mutations. Moreover, assembling them into a commercial kit is another option for the rapid and simple isolation of exosomes from small volumes of different samples, which makes these devices ideal for research and clinical settings. 

One more aspect that plays an essential role in the reliability of diagnostic techniques is quality assurance and quality control. The next steps need to ensure that the process of exosome isolation in the clinical setting is repetitive, rapid, easy to handle, and cost-effective, with a low error rate, high recovery yield, and consistently high sensitivity and specificity. Some companies have recommended a pre-clinical exosome isolation kit (like SBI’s Exoquick-CG^®^) that requires validation through clinical trials and compatibility recognition with pathology reports. However, there is not a gold standard for exosomes’ detection and analysis. To date, no approved exosome isolation procedure has been introduced for the clinical setting. The future “golden standard” to reach these milestones in microfluidics supported exosome-based disease diagnostic and monitoring will most probably combine two or more separation methods.

## 7. Conclusions

Microfluidic devices were introduced to improve the methods for exosomes’ detection and isolation by facilitating their immunological separation, sieving, and trapping. This makes them highly attractive targets for the detection and analysis of diseases. As a number of studies have demonstrated, the specific proteins and nucleic acids, as the exosomes’ “cargo”, could be an important source of biomarkers extracted from biofluids [93,96,99,101,116,175]. The RNA analysis of the exosomes from liquid biopsy samples could complement the information coming from classical DNA analysis and clarify specific processes, and eventually lead to personalized medicine. Taking into account such aspects, a liquid biopsy using microfluidic systems may also turn out to be a key strategy for developing personalized targeted therapy and disease monitoring. The main scope of the research for microfluidic devices will be the clinical translation and how to overcome the challenges faced on the way towards the clinical application of exosomes as biomarker sources. The various microfluidic LOC platforms that have emerged try to increase the sensitivity of the isolation process and to further facilitate the in situ analysis. This is in line with the current trend and demand for realizing ‘smart’ systems with increased functionality and ease of use at the POCT, even by non-specialists. Furthermore, the realization and standardized adoption and usage of such advanced equipment could be the first step towards personalized healthcare. It may also bring closer the more distant goal of realizing a machine for the holistic separation and analysis of all liquid biopsy components in order to ultimately provide highly efficient predictive medicine for actual personalized diagnosis and treatment. The intricacy and difficulty of clinical trials and validation will also need to be overcome in order to pave the way to the wide acceptance of such equipment by medical practitioners worldwide. Furthermore, the ultimate clinical challenge is to perfect the technology, increase the dynamic range of the platforms, and produce cost-effective specific and sensitive devices for personalized medicine.

## Appendix A

**Table A1:** The most common microfluidic approaches for exosomes’ isolation.

Principle for Isolation	Technology	Sample Used (Most of the Platforms Can Process Cell Culture Derived Exosomes)	Performance	Drawbacks	Application for Detection and Analysis	Clinical Potential		Source
Immunoaffinity (targeted marker)	**ExoChip:** functionalized CD63 multi-chamber/channel platform	serum–human origin	isolated size ~30–300 nm; Isolation throughput 4 μL·min^−1^; isolation capacity 400 μL	no multiplexing	detection via fluorescent staining and conventional plate reader; LOD 0.5 pM fluorescence sensitivity	CD63(+) pancreatic cancer-derived exosomes; extract total RNA		[99]
Immunoaffinity (targeted marker)	**iMEX and Nano-iMEX:** Y-shaped microposts with (CD81) nanostructured coating; nanoshearing chip, electrohydrodynamic flow-assisted immunocapture	plasma (10× dilution)–human origin	Isolated size < 150 nm; isolation throughput 0.05 μL·min^−1^; isolation capacity 20 μL; high sensitivity	-	improved capture and inline detection efficiency ~50 exosomes/μL; on-chip isolation-cum-detection via singleplex ELISA-based fluorescence imaging	overall levels of CD9 (+), CD81 (+), EpCAM (+), and CD63(+) ovarian cancer-derived exosomes; other markers: CD24, CA125, HER2, MUC18, and EGFR		[100] [176]
Immunoaffinity (targeted marker)	**nPLEX** (nano-plasmonic exosome-sensor): functionalized (CD24, CD63, EpCAM) gold surface with nanohole arrays.	ascites fluid (0.2 μm filter)–human origin	isolated size ~20–260 nm; isolation throughput 8.3 μL·min^−1^; isolation capacity 150 μL; high sensitivity recovery	-	portable simultaneous isolation and surface plasmon resonance (SPR)-based multiplexed detection; LOD: 1000 - 3000 exosomes and subpopulation	ovarian cancer without sample pretreatment (EpCAM, CD24, CD63); parallel detection of 12 potential exosomal markers: EpCAM, CD24, CA125, CA19-9, HER2, MUC18, EGFR, CLDN3, CD45, CD41, D2-40		[110] [177]
Immunoaffinity	**RInSE:** rapid inertial solution exchange; for continuous isolation of affinity-capture (EpCAM) microbeads and flow cytometry detection	blood (RBC lysis, centrifugation, incubation with capture beads and label)–human origin	isolated size 30–120 nm; isolation throughput 70 μL·min^−1^; isolation capacity–continuous flow	-	detection flow cytometry; LOD not available;	breast cancer-derived CD81 in EpCAM (+) exosomes		[154]
Immunomagnetic	**Immuno-magnetic exosome RNA (iMER)** immuno-magnetic chip: isolation using immunomagnetic (EGFR) microbeads	serum (0.8 μm filter)–human origin	isolation sensitivity > 93%; isolated size ~100 nm; isolation throughput 4 μL·min^−1^; isolation capacity 100 μL; on-chip capturing for RNA analysis	enrichment is dependent on surface marker	high efficiency and integrated exosomal RNA analysis: exosomes’ lysis and RNA capture; analysis of intra-vesicular protein and mRNA via on-chip RT-qPCR	glioblastoma treatment monitoring via exosomal mRNA [EPHA2, EGFR, PDPN, MGTM, APNG in EGFR (+) and EGFRvIII (+)];		[111]
Immunoaffinity	**ExoSearch:** continuous capture on immunomagnetic (CD9) microbeads	plasma–human origin	isolation sensitivity 72%; isolated size 50–250 nm; isolation throughput 0.8 μL·min^−1^; isolation capacity 20 μL		detection via fluorescently-labelled CA-125-, EpCAM-, and CD24-antibodies for on-chip fluorescence imaging; LOD: 750 exosomes/μL	ovarian cancer CA-125, EpCAM, and CD24 in CD9(+)-derived exosomes;		[101]
Immunoaffinity - microbead-based immunocapture	mobile exosome detector (**μMED**) with microbeads of different sizes	mouse serum	isolation sensitivity: 1 × 10^7^; isolated size ≈117 nm; isolation throughput ≲10/3 μL·min^−1^; isolation capacity ~100 μL; successfully reflected the different status of neurons; simple operation	limited sensitivity and accuracy; exosomes’ enrichment	ELISA-based optofluidic detection via smartphone; LOD: 10,000 exosomes/μL·mL^−1^	ELISA-based optofluidic detection of GluR2 in CD81(+) exosomes to detect the level of cortical neurons with and without injuries; other markers detected: EGFR, PDGFR-alpha, PDPN, ElphaA2, EGFRcIII6, IDHIRI32H, HSP90, CD41, and MHCII		[113]
Immunoaffinity	capture on functionalized (CD9, HER2) gold electrodes enhanced by nanoshearing	serum–human origin	nanoshearing enhanced capture; isolated size ~30–350 nm; isolation throughput 4.2 μL·min^−1^; isolation capacity 500 μL	data on isolation sensitivity not available	nanoshearing enhanced detection and ELISA-based colorimetric sensing; LOD: 2760 exosomes/μL	HER2(+) and CD9(+) breast cancer-derived exosomes;		[153]
Immunoaffinity	functionalized CD9 and CD63 gold surface	serum–human origin	isolated size ~30–300 nm; isolation throughput 5 μL·min^−1^; isolation capacity 250 μL	isolation sensitivity: NA	SPR-based specific detection; detection limit ~2070 exosomes/μL	HER2(+) and CD9(+) or CD63(+) breast cancer-derived exosomes		[178]
Immunomagnetic	on chip capture on immunomagnetic beads	plasma pre-mixed with capture beads–human origin	isolation sensitivity: low; isolated size ~40–150 nm; isolation throughput 2 μL·min^−1^; isolation capacity ~30 μL	concertation on-chip exosomes 100 times lower than the recovery from centrifugation	on-chip cell lysis and protein capture, ELISA-based intravesicular protein chemifluorescent analysis; detection limit: 0.281 pg·mL (IGF-1R); 0.383 pg·mL (p-IGF-1R);	phenotyping of common exosomal and tumor-specific markers; type 1 insulin growth factor receptor (IGF-1R and p-IGF-1R) in EpCAM(+) non-small-cell-lung cancer-derived exosomes		[109]
Membrane-based filtration	Exodisc: double filtration and centrifugation	urine–human origin	isolation sensitivity: 95%; isolated size ~20–600 nm; isolation throughput 36 μL·min^−1^; isolation capacity 1000 μL; high purity and dual-membrane filtration	might be prone to clogging; uniform membrane pore fabrication might be difficult; co-isolation with larger vesicles	ELISA-based colorimetric detection	overall levels of CD9 and CD81 urinary bladder cancer-derived exosomes		[179]
Membrane-based filtration	**ExoTIC:** Exosome total isolation chip	biofluids: plasma, urine, lavages	high purity and low-cost assembly; ~4–1000 fold higher yield than ultracentrifugation	fabrication of uniform multiple nano-sized pores may be challenging	EVs size-based detection and miRNA extraction for analysis	genomic, transcriptomic, and proteomic signatures for cancer		[180]
Viscoelastic flow	continuous viscoelasticity-based and field-free microfluidic sorting chip	fetal bovine serum; cell culture	separation efficiency- ~80%; label-free continuous separation; isolated size <200 nm; isolation throughput 10/3 μL·min^−1^; isolation capacity 100 μL	the poly(oxyethylene) (PEO) polymer used in the process residue might be present in retrieved EV samples: the compatibility of PEO with the sample and the effect on the downstream analysis is unknown	future validation with clinical samples required	proteomics, gene analysis	[181]	
Membrane-based filtration	double filtration chip	cell culture medium and urine (centrifugation, 0.22 μm filter)–human origin	separation efficiency—~74–81%; isolated size ~155 nm; isolation throughput 33 μL·min^−1^; isolation capacity 8000 μL; high purity	clogging especially when pore sizes are small-fabrication of 30 nm pore	ELISA-based colorimetric detection on chip	overall levels of CD63(+) urinary bladder cancer-derived exosomes	[160]	
